# High acceptability of a newly developed urological practical skills training program

**DOI:** 10.1186/s12894-015-0084-8

**Published:** 2015-09-04

**Authors:** Anna H. de Vries, Scheltus J. van Luijk, Albert J. J. A. Scherpbier, Ad J. M. Hendrikx, Evert L. Koldewijn, Cordula Wagner, Barbara M. A. Schout

**Affiliations:** Department of Urology, Catharina Hospital, Eindhoven, The Netherlands; Academy of Post-graduate Education, Maastricht University Medical Centre, Maastricht, The Netherlands; Faculty of Health, Medicine and Life Sciences, Maastricht University Medical Centre, Maastricht, The Netherlands; Department of Public and Occupational Health, EMGO Institute for Health and Care Research, Amsterdam, The Netherlands; Netherlands Institute for Health Services Research (NIVEL), Utrecht, The Netherlands; Department of Urology, St. Antonius Hospital, Nieuwegein, The Netherlands

## Abstract

**Background:**

Benefits of simulation training are widely recognized, but its structural implementation into urological curricula remains challenging. This study aims to gain insight into current and ideal urological practical skills training and presents the outline of a newly developed skills training program, including an assessment of the design characteristics that may increase its acceptability.

**Methods:**

A questionnaire was sent to the urology residents (n = 87) and program directors (n = 45) of all Dutch teaching hospitals. Open- and close-ended questions were used to determine the views on current and ideal skills training and the newly developed skills training program. Eight semi-structured interviews were conducted with 39 residents and 15 program directors. All interviews were audiotaped, fully transcribed, and thereafter analyzed.

**Results:**

Response was 87.4 % for residents and 86.7 % for program directors. Residents appeared to be still predominantly trained ‘by doing’. Structured practical skills training in local hospitals takes place according to 12 % of the residents versus 44 % of the program directors (*p* < 0.001). Ideally, residents prefer to practice certain procedures on simulation models first, especially in endourology. The majority of residents (92 %) and program directors (87 %) approved of implementing the newly developed skills training program (*p* = 0.51). ‘Structured scheduling’, ‘use of peer teaching’ and ‘high fidelity models’ were indicated as design characteristics that increase its acceptability.

**Conclusions:**

Current urological residency training consists of patient-related ‘learning by doing’, although more practice on simulation models is desired. The acceptability of implementing the presented skills-training program is high. Design characteristics that increase its acceptability are structured scheduling, the use of peer teaching and high fidelity models.

**Electronic supplementary material:**

The online version of this article (doi:10.1186/s12894-015-0084-8) contains supplementary material, which is available to authorized users.

## Background

In present time, training outside the patient is widely accepted and several studies have shown that urological skills can be improved by simulation training [1, 2]. The main advantage of training on simulators is that the patient-related learning curve can be shortened without compromising patient safety. In addition to the classical master-apprentice type of training (see one, do one, teach one), new simulation curricula are required due to the evolution of medical technology, the increasing number of minimally invasive procedures that urologists need to master, the decreasing number of patient-related training hours and patient safety issues [3–7].

Several studies have been conducted on how to develop simulation programs. Ahmed et al. concluded that ‘proficiency-based curricula with well-structured endpoints and objective tools for validating proficiency are crucial in developing a simulation program’ [8]. Sweet et al. emphasized the value of the backward design principle of Wiggins and McTighe [9]. According to this principle, the purpose and learning outcome must be determined first, after which learning objectives are established by working backwards from the desired outcomes.

Within surgery, several practical skills training curricula have been implemented and validated, such as the American College of Surgeons/Association of Program Directors in Surgery surgical skills curriculum and the Fundamentals of Laparoscopic Surgery [10–12]. For urology, only a limited number of urological practical skills training curricula have been developed [13, 14]. Moreover, their structured implementation remains challenging. Possible obstacles in the implementation of a skills curriculum in surgical residency programs are issues like limited personnel, considerable cost and resident working hour restrictions [15].

In the current Dutch urological curriculum, residents are obliged to attend a number of national practical skills courses. However, reports of the Dutch inspection of health services pointed out that residents’ knowledge of local medical technology is not optimal [16]. The outline for the Dutch Urology Practical Skills (D-UPS) training program was designed to provide residents and program directors with a structured training program for urological basic skills, including pre-test, procedural steps, simulator training, pitfalls and evaluation. One of the first steps in the development and implementation of a new curriculum is the establishment of acceptability [9]. To enhance this aspect, our implementation strategy included the early involvement of residents and program directors in the development of the program, prior to its validation and implementation.

This study presents the outline of the newly developed D-UPS program and aimed to answer the questions: ‘How do residents currently and ideally learn their practical urology skills?’ and ‘Which design characteristics may increase the acceptability of urological practical skills training programs such as D-UPS?’

## Methods

### Development of the Dutch Urology Practical Skills training program

The D-UPS program was designed using the backward design principle of Wiggins and McTighe [17]. The program combines the acquisition and rehearsal of basic theoretical knowledge, based on theory derived from the national courses and expert input, with practical training of basic urological skills and techniques. The first step in developing each specific training session was a Training Needs Analysis (TNA) [18, 19]. In the TNA, procedural steps were identified, potential pitfalls analysed and learning objectives defined [20]. Subsequently, a suitable simulator was selected (Training Media Specification, TMS), with a preference for low fidelity models to limit the costs and simplify logistics.

The D-UPS program was designed by the national project group ‘Training in Urology’ in collaboration with the Dutch Association of Urologists. In the final development of the program, the opinions of residents and program directors were considered.

### Study design

In this mixed-method research design, we used a questionnaire to collect quantitative data and semi-structured focus group interviews to collect qualitative data.

### Questionnaire

The questionnaire was developed by a multidisciplinary team, consisting of an educationalist (AS) and two experts in urology (BS, AH). The questionnaire contained nine questions or statements rated on a 5-point Likert scale (1 = disagree, 5 = agree), five open-ended questions, one yes/no question and one multiple-choice question. Three questions focused on demographics, three on the participants’ opinions on current practical skills training, and ten on the D-UPS program, e.g. positive endpoints and expected difficulties in future implementation. The full version of the questionnaire is added in Additional file 1, including the definitions of various expected positive endpoints.

Between April 2011 and December 2011, the questionnaire was sent to all 87 Dutch urology residents and 45 program directors in the 25 teaching hospitals, using the online program Survey Monkey (http://www.surveymonkey.com).

### Interviews

For the semi-structured focus group interviews, a topic list was developed by a multidisciplinary team, consisting of an educationalist (AS) and two experts in urology (BS, AH). The topic list consisted of three main themes: 1) current way of learning practical skills, 2) ideal way of learning practical skills, and 3) respondents’ opinions on the design characteristics of the D-UPS program in relation to its acceptability (Additional file 2).

All residents and program directors in the Netherlands (n = 132) were invited by email (BS) to participate in an interview. Those residents and program directors that responded positively to this electronic invitation were divided into groups based on their geographic distribution. Between March and December 2011 the interviews were conducted in five different teaching hospitals across the Netherlands. The interviews were moderated by an independent expert in medical education (SvL). Besides the moderator, one researcher was present to make field notes (BS or AH). Before the interview, participants received one page of information on the content of the D-UPS program. Interviews continued until no new themes emerged. Needed number of interviews was based on saturation of information.

### Data analysis

Questionnaire data were graphically displayed using frequency figures. Differences in categorical variables between groups were analysed using the Chi-square test. A p-value <0.05 was considered statistically significant. Analyses were performed using the Statistical Package for Social Sciences version 20.0.

Interviews were audio-recorded and transcribed verbatim by an independent company. Subsequently, transcripts were imported into a software program for qualitative data analysis (Atlas.ti version 7). The transcripts were thematically coded by the principal researcher (BS) using a predefined coding scheme based on the three main themes described earlier. To enhance interobserver reliability, 25 % of transcripts were independently coded by a second researcher (AdV). Discrepancies in initial coding between the two researchers were discussed until consensus was reached, and a final coding scheme was established. Thereafter, all interviews were summarized using the final coding scheme. The responses were categorized into the three themes. Finally, quotes were selected to illustrate findings.

### Ethical aspects

Ethical approval was sought from the Catharina hospital’s research and ethics committee. Since patients or patient data were not involved in this study, they ruled that ethical approval was not required according to the Dutch Medical Research (Human Subjects) Act. All included residents and program directors volunteered to participate and anonymity and confidentiality was guaranteed. Informed consent with assurance of anonymity was obtained at the start of each interview.

## Results

### Design characteristics of the D-UPS program

The D-UPS program features mandatory training sessions of one hour per week in the local hospital setting for junior and senior residents. The program starts with the implementation of eight training sessions of basic urological skills that will be yearly repeated, namely: ‘ultrasound of kidney and bladder’, ‘ultrasound of prostate’, ‘acute penile pathology’, ‘basic laparoscopy’, ‘electro surgery’, ‘mid urethral sling’, ‘transurethral resection of the prostrate’, and ‘flexible ureterorenoscopy’. After these training sessions have been validated, the next eight sessions will be implemented, consisting of more advanced urological skills (e.g. pyelumplasty).

**Fig. 1:**
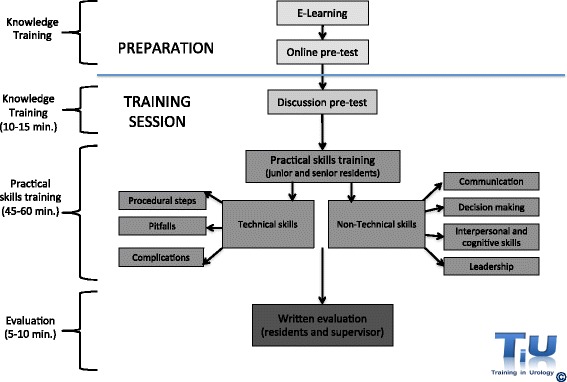
General outline of the training sessions of the D-UPS program

In preparation for each training hour residents have to study the obligatory theory, based on theory derived from the national courses and expert input, and take a formative online test that consist of approximately ten multiple choice questions. At the start of the training session, the results of this test are discussed, before all procedural steps, pitfalls and non-technical skills of the relevant procedure are trained in a non-patient-related setting under the supervision of an experienced urologist. As residents progress in their specialist training, peer teaching becomes more important, lifting the training sessions to a higher level for senior residents and preparing them for their future role as educators. Each session ends with an evaluation of satisfactory and unsatisfactory aspects of the training. Figure 1 presents the general outline of the training sessions. As an example of the actual content of the training, a summary of the training session ‘basic laparoscopy’ is presented in Additional file 3. The laparoscopic tasks used in this training are derived from the validated European Basic Laparoscopic Urological Skills Exam [21].

### Questionnaire and interviews

The response rate to the questionnaire was 87.4 % for residents and 86.7 % for program directors, representing all the 25 Dutch teaching hospitals. Interviews were conducted with five groups of residents (n = 39) and three groups of program directors (n = 15) from 20 different teaching hospitals. The median number of participants per interview was 6 (range 4–11). Interviews lasted a median of 53 min. (range 39–73).

### Current and ideal practical skills training in general

#### Questionnaire-results on general practical skills training

The results of the questionnaire revealed that some form of structured, practical skills training currently takes place in local teaching hospitals according to 12 % of residents versus 44 % of program directors (*p* < 0.001, chi-square test). The frequency of practical skills training sessions varied per hospital, from once a week to once every six months, and training was mostly provided by one of the staff urologists. Residents and program directors who reported to have some form of practical skills training in their hospital mentioned training in laparoscopy as the main practical skills training (80 % and 59 % respectively). Additionally, they mentioned training in sonography, general tips in surgical procedures, vasectomy and circumcision.

#### Interview-results on general practical skills training

All the interviewed residents and program directors stated that currently residents learn their practical skills ‘by doing’. First they observe and then they do it themselves, step-by-step, with instructions from a supervising urologist. *‘See one, do one, teach one. When you feel competent, and the program director feels the same way, they let you go.’ (resident).*

The majority confirmed the presence of a skills lab in their teaching hospital. However, only in two hospitals were these skills labs used on a regular basis. Training should be scheduled, since voluntary training does not take place due to residents’ busy schedules. Residents stated that materials in the skills lab are often lacking or in bad condition. The majority of residents considered it desirable to practice certain procedures first on a suitable simulation model, especially in endourology and laparoscopy. They would like to practice in a non-patient-related setting more often. *‘The question is: would I have preferred to practice on a simulator? The answer is yes, absolutely. But this was not an option.’ (resident)* Program directors shared this opinion, provided that adequate simulation models were available for training.

### Quality of the newly developed D-UPS program

#### Questionnaire-results on opinions about D-UPS

**Fig. 2:**
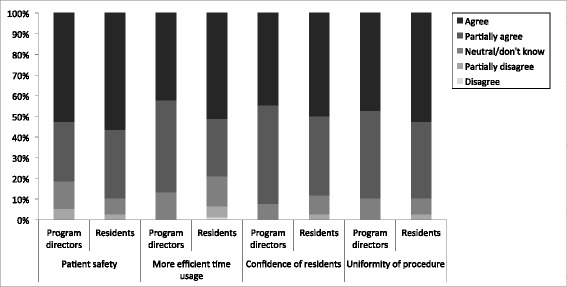
Views of residents and program directors on expected positive endpoints in implementation of the D-UPS program. There were no significant differences in opinions of residents versus program directors regarding expected effects on patient safety (*p* = 0.35), time efficiency in the OR (*p* = 0.44), self-confidence of the residents (*p* = 0.75), and uniformity of procedure (*p* = 1.0)

**Fig. 3:**
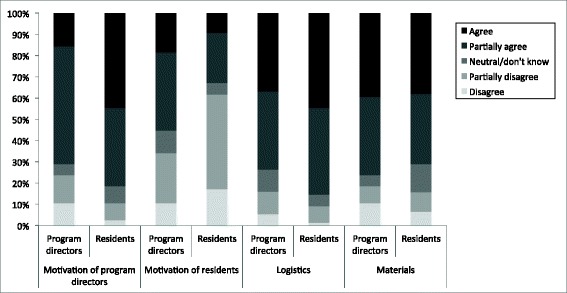
Views of residents and program directors on expected difficulties in implementation of the D-UPS program. There were no significant differences in opinions of residents versus program directors regarding expected difficulties in motivation of residents (*p* = 0.23), logistics (*p* = 0.13), and materials (*p* = 0.66). *The majority of residents believed motivation of the program directors would be a difficulty in the implementation of the proposed training program, in contrast to the program directors (*p* = 0.02)

The majority of residents and program directors considered the D-UPS program to be a useful addition to present education (92 % and 87 % respectively, *p* = 0.51). They expected structured practical skills training to have a positive effect on patient safety, time efficiency in the OR, self-confidence of the residents and uniformity of actions (Fig. 2). There were no significant differences in opinions between residents versus program directors. The main expected difficulties in the implementation of the D-UPS program (Fig. 3) were logistics and lack of motivation of the program directors. Significantly more residents than program directors expected the motivation of the program director to be a problem (*p* = 0.02).

#### Interview-results on opinions about D-UPS

The interviews revealed that residents expected the D-UPS program to provide them with knowledge of instruments and to make them more familiar with procedural steps. They also revealed another challenge, which was adaptation of the training level to senior residents. Moreover, the interviews revealed further details concerning design characteristics in relation to the acceptability of the program. Residents and program directors were enthusiastic about the design of the D-UPS, because it would create a nationwide uniform foundation of basic urology techniques. The use of peer teaching was noted as important for residents’ future role as educators and for making the training sessions easily accessible. Some criticism was expressed on the time frame of the training sessions. Various residents and program directors indicated that one hour might not be sufficient for training certain skills, and one afternoon per month was suggested as an alternative. Moreover, residents and program directors stressed structured scheduling of the training sessions as an important condition for successful implementation of the D-UPS program. Residents indicated to be keen on training practical skills on simulation models, particularly in endourology. However, the use of realistic, high fidelity simulation models was emphasized as an important condition for successful implementation. In summary, according to residents and program directors, the design characteristics that could increase the acceptability of urological practical skills training programs were structured scheduling, the use of peer teaching and high fidelity models.

## Discussion

In this study, we aimed to gain insight into current and ideal Dutch urological skills training and presented the outline of the D-UPS program, including the assessment of design characteristics that may increase its acceptability. The results of this study show that Dutch residents in urology currently learn their practical skills ‘by doing’, according to the classic master-apprentice model. Ideally, they would prefer to practice certain procedures on simulation models first, especially in endourology. The acceptability of implementing the newly developed D-UPS program is high. Residents and program directors think this program would provide all residents in urology with a nationwide uniform foundation for training urological techniques. Design characteristics that increase acceptability of the D-UPS/related practical skills training programs are discussed in the next paragraphs.

One of the expected difficulties in implementing the skills training program was ‘materials’. Residents and program directors expressed the belief that practical skills training is only useful if residents practise on realistic, i.e. high fidelity models. This is contradictory to the present outline of the D-UPS program, in which low fidelity models are preferred. In the decision of which simulator to use for skills training it is of paramount importance that the simulator can serve the goal of training. In the development of the D-UPS program, first the learning objectives for training a certain skill were defined and subsequently a suitable simulator was sought. If possible the choice was for a simulator of low fidelity. This was not only to limit the cost, but also to simplify logistics and because for certain basic skills no high fidelity models are available. In the literature it is confirmed that, especially for training basic skills, low fidelity simulators can be of great value. Matsumoto et al. compared the effectiveness of a strictly didactic training in ureteroscopy with training on a low fidelity model and on a high fidelity model [22]. They showed that training on the low fidelity model had the same degree of benefit as training on the high fidelity model, and both had a significantly higher degree of benefit than the didactic session alone. Since the first eight training sessions focus on basic urological procedures, low fidelity simulators could be suitable. However, when it comes to training more advanced skills sometimes high fidelity simulators, e.g. virtual reality simulation, will be needed. For successful implementation of practical skills training using low fidelity models, it will be of great importance that residents and program directors understand the value of these training models. McDougall et al. designed a 4-year curriculum for urology residency training, with frequent training sessions using mainly low fidelity models [14]. Although this study included only 8 residents so far and evaluation is ongoing, initial results are encouraging. Most participants stated that this 4-year curriculum provided a better learning experience than the curriculum without structured skills training. Furthermore, while residents and program directors in our study expected one-hour training sessions to be insufficient for some parts of practical skills training, McDougall and colleagues found that acceptance of a weekly hour of training was high [14]. In their study, the majority of residents indicated that one hour of training was sufficient and provided new clinical information.

Another important expected obstacle, according to residents and program directors in our study, was the logistic integration of practical skills training into the working week. Structured scheduling was suggested as a condition for successful implementation. The importance of scheduling training sessions and making them obligatory was emphasized by Chang and colleagues, who examined the effectiveness of voluntary training in a simulation laboratory as part of the surgical curriculum [23]. They showed that voluntary use of a surgical simulation laboratory resulted in minimal participation in the curriculum.

Another expected difficulty in implementation was motivation, in particular the motivation of program directors. This concern is in line with the findings of Stefanidis et al., who described the implementation of a proficiency-based laparoscopic skills curriculum in a general surgical residency program and found that this can only be achieved successfully if dedicated faculty and scheduled training time are ensured [24]. Hence, one of the key success factors for implementation is motivating program directors for their educational role in urology skills training programs.

A remarkable finding was the significant difference in views on the current availability of structured practical skills training in the local teaching hospitals. This was mentioned as current practice by 12 % of residents versus 44 % of program directors. A possible explanation for this difference could be that residents and program directors have different perceptions of the definition of practical skills training, or that some of the residents started their residency only recently, and might not yet have been involved in practical skills training.

To our knowledge, the D-UPS program would be the first curriculum in Europe that provides yearly repetitive practical skills training in the local hospital setting, including the use of the local equipment. The first step in the development and implementation of a new curriculum is the performance of training needs analysis and the establishment of acceptability, which was evaluated in this study. Although the results of this study describe the Dutch situation, which limits generalizability, the outline of the D-UPS program could serve as a blueprint for skills training in other surgical specialties in the Netherlands. Moreover, extrapolation to European countries would be possible, especially those countries with similar residency programs, since up till now there have been limited initiatives for non-patient related skills training curricula.

Where possible, existing validated simulation training is incorporated in the D-UPS program, to avoid duplication and expense. For example, the tasks used in the basic laparoscopy training of the D-UPS program are derived from the validated European Basic Laparoscopic Urological Skills program [21]. Other possibilities should be further explored.

We acknowledge that validation of the curriculum is of paramount importance in the process of innovating educational programs. However, this is a multi-year process and is considered to be the endpoint of the implementation process. In the process towards this validation it is important to inform colleagues in the field of curriculum development regarding the ongoing developments, since they might profit from the outline of this program an our findings on design characteristics that increase the acceptability of implementing practical skills training in a non-patient-related setting.

The use of a questionnaire and interviews is relatively subjective and might have led to socially desirable answers. To counter this effect, the interviews were moderated by an independent educational expert, and anonymity was guaranteed. Furthermore, residents and program directors were interviewed in separate groups to ensure freedom and safety in expressing opinions. As in any qualitative study, investigator objectivity is a limitation [25]. This issue was countered by having 25 % of the transcripts coded by two researchers separately.

## Conclusions

Current urological residency training consists of patient-related ‘learning by doing’. Structured, practical skills training takes place in a minority of teaching hospitals. Ideally, residents and program directors would welcome more practice on simulation models. Design characteristics that increase the acceptability of implementing a skills training program are structured scheduling, the use of peer teaching and the use of high-fidelity models. The acceptability of implementing the presented skills training program is high.

## Additional files

Additional file 1:
**Questionnaire.** (PDF 97 kb) Additional file 2:
**Topic list semi-structured focus group interviews.** (PDF 75 kb) Additional file 3:
**Content basic laparoscopy training.** (PDF 6710 kb)

## Abbreviations

OR: Operating room
D-UPS program: Dutch Urology Practical Skills program
TNA: Training needs analysis
TMS: Training media specification
