# Supplementary material for: High acceptability of a newly developed urological practical skills training program
Source: BMC Urol. 2015 Sep 4;15:93. doi: 10.1186/s12894-015-0084-8 (PMC4560076; doi:10.1186/s12894-015-0084-8)
Supplement: Additional file 1: — Questionnaire. (PDF 97 kb) [file 12894_2015_84_MOESM1_ESM.pdf]

Additional file 1. Questionnaire

DEMOGRAPHICS

All data will be processed anonymously!

1. Name
2. Teaching hospital
3. Year of residency training (for residents)

CURRENT RESIDENCY TRAINING PROGRAM

4. Is non-patient related practical skills training (diagnostic and operative skills) a structured part of residency training in your teaching hospital?
□ Yes
□ No

If Yes:

5. Who provides / supervises this structured local non-patient related practical skills training? (Multiple answer options possible)
□ Program director
□ Staff urologist
□ Resident
□ External people

5. Which practical skills are trained and what is the frequency of this practical skills training?

____

D-UPS PROGRAM

6. Implementation of the D-UPS program would be a useful addition to present residency training
□ Agree
□ Partially agree
□ Neutral / don't know
□ Partially disagree
□ Disagree

7. Expected positive effects of the implementation of the D-UPS program would be:

	Agree	Partially agree	Neutral / don't know	Partially disagree	Disagree
Patient safety*					
More efficient time usage in the operating room*					
Increase in self-confidence of residents					
Uniformity of actions of residents					

Others and / or explanation

____

* Definition of endpoints:
- Patient safety: the prevention of errors and adverse effects to patients associated with health care
- More efficient time usage in the operating room: shorter procedural time due to a shortened patient related learning curve

8. Expected difficulties in the implementation of the D-UPS program would be:

	Agree	Partially agree	Neutral / don't know	Partially disagree	Disagree
Motivation of program directors in participation and organization of the training sessions					
Motivation of residents in participation and organization of the training sessions					
Logistics of one hour of training per week					
Availability of simulation models and materials					

Others and / or explanation

____

9. Procedures that should definitely be incorporated in the D-UPS program:

____

Thank you very much for your time and participation!!

Place for remarks / general comments

____

____
