# Supplementary material for: High acceptability of a newly developed urological practical skills training program
Source: BMC Urol. 2015 Sep 4;15:93. doi: 10.1186/s12894-015-0084-8 (PMC4560076; doi:10.1186/s12894-015-0084-8)
Supplement: Additional file 2: — Topic list semi-structured focus group interviews. (PDF 75 kb) [file 12894_2015_84_MOESM2_ESM.pdf]

Additional file 2. Topic list semi-structured focus group interviews

Duration: one hour
Bold = must be discussed!

DEMOGRAPHICS

1. Ask if it is possible for everybody to switch of their mobile phone and leave their pager at the secretary.
2. **Ask permission for recording the interview.**
3. **Personal details: ask for name, age, year of residency training / number of years employed as program director, name of hospital.**

CURRENT AND IDEAL WAY OF LEARNING PRACTICAL SKILLS

4. **How do you currently learn your diagnostic and operative practical skills (master-apprentice / skillslab)?**
5. How many times have you been supervised before you started to perform procedures independently / without supervision?
6. How do you experience the current master apprentice type of training practical skills?
7. What are good aspects of the current master apprentice type of training practical skills?
8. What are points for improvement of the current master apprentice type of training practical skills?

DESIGN CHARACTERISTICS OF D-UPS PROGRAM IN RELATION TO ITS ACCEPTABILITY

Short written introduction regarding the outline of the D-UPS program.

9. **What do you think of these plans?**
10. What would be the difficulties regarding these plans?
11. What should be the role of the program director within the D-UPS program?
12. What should be the role of the resident within the D-UPS program?
13. **Is there a skills lab present in your hospital where you can train on simulation models?**
14. **Which urological skills would you most prefer to train?**
15. Do you think that also non-technical skills, such as team training, should be incorporated in the D-UPS program?

FINAL REMARKS

- Thank you for your participation.
- An email including the main points of the interview will be send out for your final consent.
- A quantitative survey will follow within the next weeks. We kindly ask you to fill out this questionnaire.
