# Supplementary material for: High acceptability of a newly developed urological practical skills training program
Source: BMC Urol. 2015 Sep 4;15:93. doi: 10.1186/s12894-015-0084-8 (PMC4560076; doi:10.1186/s12894-015-0084-8)

BASIC LAPAROSCOPY

Procedural steps

1. Check materials
2. Positioning patient
3. Trocar insertion
4. 3-dimensional orientation and movement
5. Usage of the camera
6. Cutting
7. Grasping
8. Tissue dissection
9. Applying appropriate traction to tissue
10. Smooth clipping
11. Solve bleedings
12. Suturing, handling needle driver

Simulator

Box trainer

Pitfalls technical skills

Counter-intuitive movement/fulcrum effect
Eye-hand coordination
Anatomical orientating
Image interpretation (2D-3D)
Tissue handling
Lack of experience in open procedure or anatomy
Orientation of position of patient with regards to position of instruments

Pitfalls non-technical skills

Lack of knowledge of materials
Insufficient preparation
Trouble shooting in material or equipment errors
Progress of procedure in case of difficulties in orientation
Teamwork operator/assistance
Risk assessment in decision to convert to open
Knowledge of anatomy (difference open and laparoscopic procedure)

Task 1: Peg Transfer

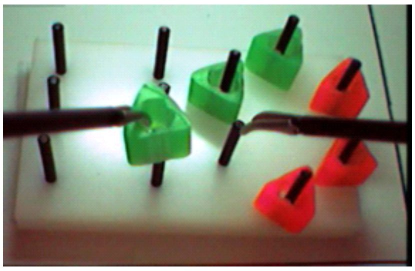

Task 2: Cutting a Circle

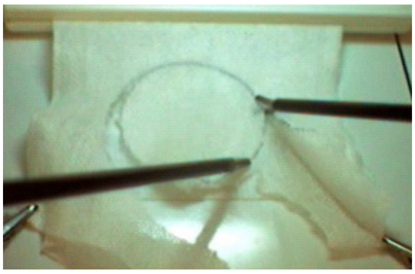

Task 3: Single knot tying

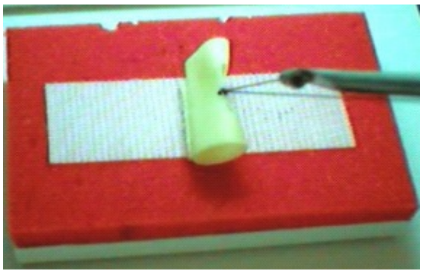

Task 4: Clip & Cut

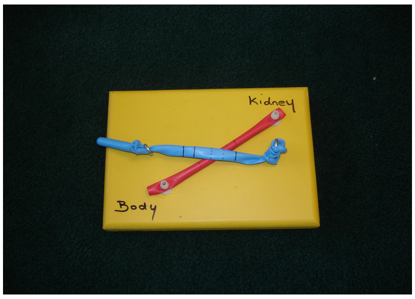

Task 5: Needle guidance

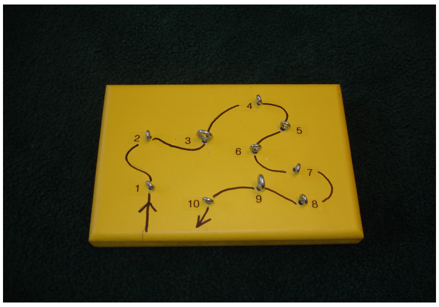

Supplement: Additional file 3: — Content basic laparoscopy training. (PDF 6710 kb) [file 12894_2015_84_MOESM3_ESM.pdf]
